# Study protocol: A cluster randomised controlled trial of implementation intentions to reduce smoking initiation in adolescents

**DOI:** 10.1186/1471-2458-13-54

**Published:** 2013-01-19

**Authors:** Mark Conner, Sarah Grogan, Rebecca Lawton, Christopher Armitage, Robert West, Kamran Siddiqi, Brenda Gannon, Carole Torgerson, Keira Flett, Ruth Simms-Ellis

**Affiliations:** 1 Institute of Psychological Sciences, University of Leeds, Leeds LS2 9JT, UK; 2 Department of Psychology, Sports and Exercise, Staffordshire University, Stoke-on-Trent ST4 2DF, UK; 3 Department of Psychology, University of Manchester, Manchester M13 9PL, UK; 4 Institute of Health Sciences, University of Leeds, Leeds LS2 9JT, UK; 5 Department of Health Sciences, University of York, York YO10 5DD, UK; 6 School of Education, Durham University, Durham DH1 1TA, UK

## Abstract

**Background:**

The current literature suggests that forming implementation intentions (simple ‘if-then’ plans) about how to refuse the offer of a cigarette may be an effective intervention to reduce smoking initiation in adolescents. This study is a pragmatic trial to test the effectiveness and cost-effectiveness of such an intervention in reducing smoking initiation in a sample of UK adolescents.

**Methods/Design:**

A cluster randomised controlled trial with at least 36 schools randomised to receive an implementation intention intervention targeting reducing smoking initiation (intervention group) or increasing homework (control group). Interventions will be conducted at the classroom level and be repeated every six months for four years (eight interventions). Objectively assessed (carbon monoxide monitor) and self-reported smoking plus smoking related cognitions (e.g., smoking intentions, attitudes, norms and self-efficacy) will be assessed at baseline and 12, 24, 36 and 48 months post baseline. Objectively assessed smoking at 48 months post baseline will be the primary outcome variable. Health economic analyses will assess life years gained.

**Discussion:**

The results of the trial will provide information on the impact of a repeated implementation intention for refusing offers of cigarettes on rates of smoking initiation in adolescents.

**Trial registration:**

ISRCTN27596806

## Background

Both in the UK and internationally, tobacco smoking continues to be an important cause of morbidity and mortality. For example, smoking related illnesses are estimated to kill over 100,000 people in the UK each year [1,2], usually later in life. Yet smoking is a behaviour that is, in general, taken up between the ages of 10 and 20 years. A variety of different research studies have supported the idea that the vast majority of smokers take up this habit as adolescents [3-5] with 40% of adult smokers having started before they reached 16 years of age [1]. The General Household Survey [6] reported that 38% of adult regular smokers took up the habit before the age of 15 years. This appears to be the case despite the fact that health promotion messages have ensured that awareness of the health consequences of smoking is now widespread, even among the young. In the UK, while the rates of regular smoking at 11 years of age are only 0.5%, this rapidly rises to 15% by 15 years of age, and then more gradually to around 20% among young adults [7]. The recent UK Department of Health [2] plan is to reduce the rates of regular smoking in 15 year olds to 12% or less by the end of 2015. Two important ways to tackle smoking-related harm are interventions to help individuals (usually adults) quit smoking and interventions to help individuals (usually adolescents) not to initiate smoking. The present paper reports the protocol for an intervention designed to reduce smoking initiation in adolescents as potentially the most effective way to reduce smoking-related harm.

A worthwhile intervention to reduce smoking initiation in the groups most likely to take up this habit (i.e., adolescents) would need to have at least three important characteristics: strong effects on reducing initiation; wide reach; and low cost. Promising initial data have been collected [8] (i.e., an explanatory trial) on the efficacy of one intervention technique that may have all of these characteristics. The study outlined here would aim to collect further data on the outcome effectiveness and cost-effectiveness of this technique in the form of a cluster randomised controlled trial (i.e., a pragmatic trial). Given the nature of the existing data in this area, this has been cast as a pragmatic trial [9] that could inform the potential roll out of this intervention for widespread use. The intervention in question is the formation of *repeated implementation intentions* about how to refuse offers of cigarettes. Implementation intentions are simple ‘if-then’ plans [10] about how to respond to environmental cues in order to help achieve a goal such as not taking up smoking, e.g., If offered a cigarette I will say ‘no thanks, I don’t smoke’. Such plans can be formed before the opportunity to act presents itself and have been found to be an effective means to change a range of behaviours [11]. Our research has shown that the repeated formation of implementation intentions about how to refuse offers of cigarettes can have a strong effect on reducing smoking initiation rates among adolescents [8,12]. In addition, this is a simple intervention that could be deployed across most schools (i.e., has wide reach) in order to help tackle smoking initiation in adolescent groups. Finally, this intervention is relatively low cost, requiring around 30 minutes per session to implement (including anti-smoking messages and completing an implementation intention questionnaire). Such an intervention could offer ‘value for money’ and be rolled out to significant proportions of the adolescent population in the UK.

Adolescence is the period during which the vast majority of smokers take up this habit in the UK [1,3]. As such it represents a key period during which to intervene. A considerable number of interventions have been tested in this age group. The vast majority of such interventions have been school-based but have met with only mixed evidence of effectiveness (for reviews see [13,14]). In part this may be attributable to lack of thorough evaluation. Nevertheless, even among high quality randomised controlled trials of school-based interventions the evidence is mixed. For example, information-based interventions have generally been ineffective, while social influence interventions have tended to show rather mixed effectiveness [13]. The most highly regarded and longest trial of this type of intervention was the Hutchinson Smoking Prevention Project which reported no evidence for effectiveness over a period of 8 years [15]. The ASSIST project is another well conducted trial carried out in the UK to evaluate a peer-led intervention to reduce smoking initiation [16]. This RCT did report support for a peer-led intervention over both a one- and a two-year period. More mixed evidence has been reported for interventions that target social competence or test multi-modal interventions [13], although there are far fewer such studies in this area. A further problem has been that many of these interventions have been quite intensive and costly to implement. For example, the Hutchinson Smoking Prevention Project [15] included a total of 65 lessons provided over a period of 8 years. Similarly, the ASSIST intervention involved two-day training events for the peer supporters. Such interventions may be costly and difficult to scale up into population level interventions that could be conducted in all schools across the UK at reasonable cost and with relative ease. In contrast, an intervention involving forming a repeated implementation intention about how to refuse offers of cigarettes would appear to be efficacious in reducing smoking initiation rates [8,12] and is also simple, easy to administer, cheap, and would appear to have the potential to be readily scalable up into a population-level intervention delivered in the vast majority of UK classrooms.

Gollwitzer [10] has defined an implementation intention as a plan of how, where and when to commit a behaviour (see [11] for a review). This type of plan establishes an ‘if-then’ link between a situation and a planned behaviour (e.g., between the offer of a first cigarette and a refusal strategy). Implementation intentions have proved to be effective yet simple means of changing a range of different health behaviours [17]. Gollwitzer and Sheeran’s [11] meta-analysis showed that across 94 independent studies in both laboratory and field settings, implementation intentions had an average effect size of d+ = 0.65, although only 6 out of the 94 studies reviewed by Gollwitzer and Sheeran [11] investigated health-risk behaviours and none examined smoking. More recently, research has begun to emerge to suggest that implementation intentions may be effective in promoting smoking cessation. For example, in two field experiments, Armitage [18,19] found that implementation intentions caused significantly more smokers to quit (up to 19% quit) compared with smokers randomly allocated to active control conditions (2% quit), suggesting that the technique has utility in this domain.

Implementation intentions have also been used in relation to reducing smoking initiation. Two studies have assessed the impact of forming an implementation intention about what to say to refuse the offer of a cigarette on subsequent smoking initiation. Higgins & Conner [12] reported that among adolescents who formed such an implementation intention (on a single occasion) 0% went on to initiate smoking in the next two months, whereas 6% did so in a control condition. Conner & Higgins [8] more recently reported the results of an explanatory cluster randomised controlled trial of repeated implementation intentions in a more appropriately powered study. Classes of children were randomly allocated to complete implementation intentions about how to refuse offers of cigarettes (intervention) or complete their homework (control) on 7 occasions between the ages of 11–12 and 13–14 years (both groups also read simple anti-smoking messages on each occasion). Research assistants delivered the intervention in classroom time to whole classrooms of children in approximately 30 minute sessions. After controlling for various known predictors of smoking initiation (e.g., gender, attitudes to smoking, friends and family smoking) and the multi-level nature of the data, rates of self-reported and objectively assessed smoking at age 15–16 years were significantly lower in the intervention compared to the control condition (d+ = 0.24 and 1.04 for self-reported smoking and objectively assessed smoking respectively). For self-reported smoking, the unadjusted reduction in smoking initiation was approximately 7% between the intervention and control conditions, while the unadjusted difference for objectively assessed smoking was approximately 10%.

### Research questions

The following research questions will be addressed in this trial:

• Can repeated implementation intentions related to refusing offers of cigarettes reduce smoking initiation rates in 11–16 year olds relative to a control group of adolescents?

• What is the cost effectiveness of such an intervention?

## Methods/Design

### Design

**Figure 1:**
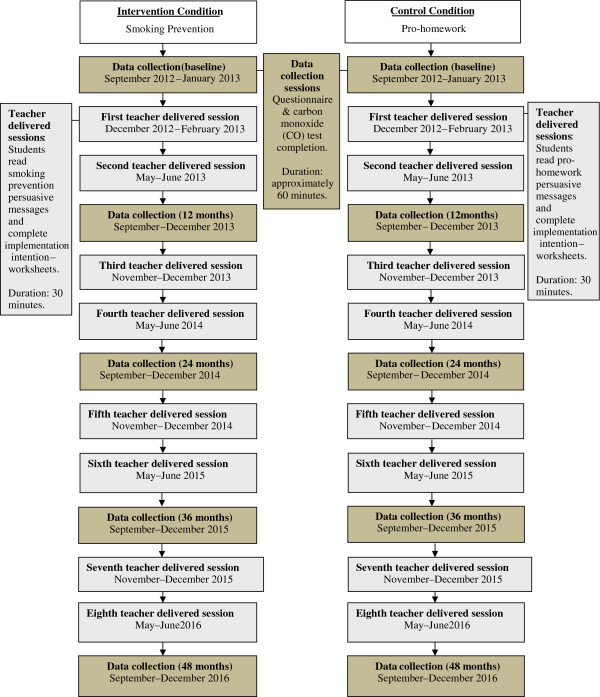
Flow Chart of Study Procedures for both Groups.

This study will use a pragmatic cluster randomised controlled trial design (see Figure 1). The unit of randomisation will be schools. Individual adolescents will be the unit of analysis although we will take account of clustering by schools.

### Sampling and recruitment of schools and adolescents

**Figure 2:**
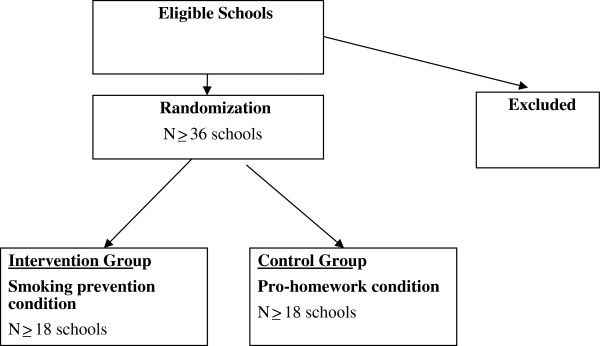
Randomization flow chart.

All secondary schools (except independent schools) in the Leeds and Staffordshire areas will be invited to take part. There will be a total of at least 36 schools recruited into the RCT (see Figure 2). We will approach secondary schools in the West Yorkshire and Staffordshire Local Education Authorities (the participating regions) and the surrounding areas to request participation in the proposed research. Through previous work and that of our collaborators we have good experience of recruiting and working with schools in both regions. Based on our previous work [8,12] we anticipate at least 50% of schools will agree to participate. Within each school all available classes in year 7 (11–12 year olds) will participate (estimated to be at least 4 classes of 30 adolescents per school).

We will ask head teachers to sign a written consent form agreeing that their schools take part in the trial and agreeing not to introduce any new anti-smoking interventions during the time period of the trial without informing the trial staff. We will seek to record carefully any anti-smoking initiatives conducted in each school during the trial period. Through the schools we will seek to obtain parental consent (an opt-out procedure) for participation in the RCT (intervention plus data collection) through letters from the participating schools.

### Study population

#### Proposed sample size

In selecting a sample size we have based our estimates on six factors: 1) the size of effect observed in our previous research; 2) the fact that school will be the unit of randomisation and adolescents will be clustered within schools; 3) a likely dropout rate based on our previous research; 4) an estimate of the intra-class correlation coefficient (ICC); 5) choice of significance level at 5% and sought power of at least 90%; and 6) a correction for imbalances in recruitment or drop-out rates across schools. Based on these values we have estimated the power of our study to detect differences between intervention and control arms of the RCT using our proposed sample sizes. In relation to effect size, our explanatory trial [8] indicated that the intervention reduced objectively assessed smoking rates from around 14% to around 4% (in unadjusted data) in 15–16 year olds. In the analysis controlling for other predictors of smoking and the multilevel nature of the data (children clustered within classes, classes clustered within schools) this equated to a large effect size with an odds ratio of 0.15 (95%CI: 0.03–0.80). We have conservatively powered the present study using a value at the upper end of this range, i.e., to detect a difference of 5% between the intervention (9% rate of smoking initiation) and control (14% rate of smoking initiation) arms at the end of the study (i.e., equivalent to an odds ratio of 0.61). In relation to dropout rates we have estimated that 85% of those who begin the study will be available to follow-up at 48 months [8]. For ICC we have estimated a value of 0.01 based on previous research [8]. In relation to significance level we have used an alpha of 0.05 using a two-sided test in order to be open to testing the possibility that the intervention actually increased smoking initiation rates. In relation to a correction factor to allow for imbalances in recruitment or dropout we estimate this will not reduce our statistical power by more than 1%.

Using these assumptions we have estimated that we require a total of 36 schools with 18 randomly allocated to the intervention arm and 18 to the control arm of the RCT. Within each school, we estimate that we need to approach at least 120 adolescents at baseline (approximately 4 classes per school). Based on an 85% recruitment rate (due to refusal to participate and non-attendance on testing days) the overall number of adolescents available for analysis will be: 0.85 * 18 * 120 = 1836 per group. The effective number of adolescents per group will be reduced further due to a design effect (i.e., adolescents clustered by school): Design effect = 1 + (m – 1)*ICC, where m is the number of participants per cluster. This yields a design effect of 2.01 and reduces the effective sample size per group to 913 (1836/2.01). This effective sample size yields a statistical power of 90.65% to detect a 5% difference in smoking initiation between the intervention (9% initiation) and control (14% initiation) arms using a two-sided test with an alpha of 0.05. Adding our correction factor for potential imbalances in recruitment would still provide a power of almost 90%. Therefore our proposed sample size is 4320 adolescents from 36 schools (18 intervention; 18 control), with approximately 120 adolescents being approached per school.

### Discontinuation criteria

Analyses will follow intention-to-treat (ITT) principles as far as possible. Therefore we will include in the analyses all schools and children initially randomised who provide data at baseline, including them for the purpose of analysis in the group originally allocated to them. To maximise the power of such analyses all reasonable and ethical steps will be taken to ensure the completeness of follow-up measures. Where data are missing we will use multiple imputation based on baseline data to replace missing data [20]. Children missing at baseline but present at one or more subsequent data collection time points will be treated as ‘other participants’ and excluded from the intention-to-treat analyses.

### Schools withdrawal

If a school wishes to withdraw from the study we will attempt to collect data on the reasons for withdrawal. We will explore with the school the possibility of using all data collected up until that point and the possibility of still collecting data from pupils at the school at the final time point in order to maximise the chance of having more complete data on our key outcome measures (an ITT principle). Provided data is available from baseline, multiple imputation will be used to replace missing data. Where no baseline data are available all data will be excluded from analyses as there will be insufficient data to appropriately perform any multiple imputation of missing values.

### Child withdrawal

Parents will have the opportunity to have their child not included in the study before commencement. Parents will also have the opportunity to withdraw their child from the study at any time point by informing the school. Further data will not be collected from children withdrawn from the study (missing data will be replaced based on multiple imputation from available data). As data will be collected anonymously, it will not be possible to withdraw data already collected. Children withdrawn from the study may not be left out of any whole class activities that form part of the intervention as to do so might involve taking the child out of the class whilst these activities were occurring.

### Interim analysis and stopping rules

Overall smoking rates in the two conditions will be computed and presented to the Independent Trial Steering Committee (TSC; and associated data monitoring committee) each year. Evidence of considerably higher rates of smoking initiation in the intervention condition compared to the control condition could be considered by the Committee as reason to stop the trial. All adverse events would also be reported to the TSC.

### Randomisation

The unit of randomisation will be schools and our sample size calculations and analyses takes account of this clustering of the data. We believe randomising by school before the intervention begins is most appropriate in order to reduce the likelihood of contamination between the intervention and control conditions (randomising by individual or classroom risks such contamination). The Trial Statistician (RW) will use a random number generator to randomly allocate schools to the control or intervention arms of the trial. Schools have requested feedback on which condition they are randomised to before giving final agreement to participate and agreeing to send out letters to parents. The trial will therefore deviate from best practice in recruiting children to the trial after rather than before randomisation. We will monitor any consequential effects on participation rates. As we hope to recruit all classes in a school year movement between classes within a school should not be a problem. However, adolescents moving school during the RCT are likely to have missing data. Of those initially participating in the study at age 11–12 years we anticipate a cumulative loss to follow-up at age 15–16 years of 15% (based on our previous research, [8]). We will make efforts to minimize drop out (e.g., attempting to ensure each school is visited on more than one day to collect smoking measures) and any potential biases attributable to drop out will be explored statistically.

### Compliance with good practice

• All statistical analyses of primary and secondary outcomes will be carried out under the guidance of the trial statistician (RW).

• HTA guidelines for missing data in RCTs will be followed [20].

• CONSORT guidelines [21,22] for presentation of results from cluster randomised trials will be followed.

• Presentation of results will conform to good practice for presentation of complex interventions [23].

• The flow of clusters and individuals through the trial, from assignment to analysis, will be presented using a flowchart, in accordance with CONSORT guidelines [21,22].

• Intra-class correlation coefficients from the multilevel analyses will be presented following good practice for cluster randomised trials.

### Ethical considerations

Ethical approval was obtained through the University of Leeds Institute of Psychological Sciences ethics committee (Reference number 12–0155). The University of Leeds will act as trial sponsor. Written informed consent will be obtained from all participating schools. The informed consent of participants’ parents will be sought by letter using an opt-out approach, i.e. parents will have the opportunity to “opt out” of the study if they do not wish their child to take part. Children will receive information sheets about the study and will have the opportunity to not complete the measures.

### Data collection methods

The primary outcome measure is smoking initiation based on objective measures at 48 months. A secondary measure will be self-reported smoking. These measures will also be assessed at baseline and at 12, 24, and 36 months post-baseline in order to explore the time periods over which the intervention might be best targeted. These data collection sessions will be completed in classroom time by the researchers and take approximately 30 minutes. Self-reported smoking and measures of cognitions (e.g. attitudes to smoking, intentions to resist smoking, self-efficacy over resisting, family smoking and friends smoking) will be collected via questionnaire completed individually but in a classroom setting. An objective measure of smoking will be completed individually outside a classroom setting. A number of measures of objective smoking are feasible. The two most common measures are breath carbon monoxide measures and saliva cotinine measures. Although saliva cotinine may be more effective in detecting occasional smoking (due to the greater half-life of 16–18 hours versus 4–6 hours in breath carbon monoxide) it also has a number of disadvantages. These include lower acceptability to adolescents, greater cost, and loss of ability to feed back the results to participants immediately. For this reason our objective measure of smoking will be obtained from breath carbon monoxide monitors (Micro+ Smokerlyzer® CO Monitor, Bedfont Scientific Limited, Kent, England). This instrument gives a measure of carbon monoxide in the breath in parts per million (ppm) accurate to within 2% based upon exhaling one breath into the device and has been adapted for use in adolescent samples. Although a number of factors influence carbon monoxide in the breath, recent smoking should significantly elevate levels. Carbon monoxide in the breath can be used as a reliable and valid measure of exposure to cigarette smoking [24], comparable in accuracy to blood carboxyhaemoglobin levels [25]. The Bedfont devices have been demonstrated to give reliable and valid assessments of smoking status [26] and have been employed by various researchers with adolescent samples [8,27]. Our secondary outcome measure, self-reported smoking, will be assessed by standard measures (as used in national surveys and our previous research; [8,28]).

### Intervention

This will be a phase III pragmatic randomised controlled trial conducted in two geographically distinct regions of England (West Yorkshire and Staffordshire) that will provide the information on which to base a decision about roll out of the intervention. The intervention involves asking adolescents to read simple anti-smoking messages and then to form an implementation intention by planning how, where, and when to resist smoking (i.e., refuse the offer of a cigarette). This intervention will be repeated every six months over a period of four years between the ages of 11–12 and 15–16 years (8 occasions; see flow chart of study procedures in Figure 1). The anti-smoking messages focus on simple-to-understand negative consequences of starting smoking and the positive consequences of remaining a never-smoker. The implementation inten-tion intervention is designed to give adolescents simple responses for how to refuse a cigarette and to link them to situations where a cigarette might be offered. Five options will be provided for *how* they could refuse the offer of a cigarette or resist the temptation to smoke (e.g., ‘No thanks, I don’t want to smoke; No thanks, I don’t want the habit; No cancer sticks for me; No thanks, smoking makes you smell bad; No, it’s bad for your health’; see [8]). Participants are required to check the options they plan to use or to write in an additional response. Similarly participants will be required to check *where* they would not smoke (e.g., ‘I will not smoke at school; I will not smoke at home; I will not smoke at a party; I will not smoke with my friends; I will not smoke if offered a cigarette’) and *when* they would not smoke (e.g., ‘I think I can make sure I don’t smoke this term’) and signal agreement with their plan not to smoke by ticking a box. The implementation intention will be presented as a paper and pencil questionnaire that adolescents read and complete in classroom time. Previous evidence has suggested that implementation intentions are more effective among individuals who are motivated to perform the behaviour [17]. Therefore combining the implementation intention with motivational messages is likely to be more effective than using implementation intentions alone. This was the combination used in our previous research [8,12]. The research has broadly followed the MRC framework for developing and evaluating complex interventions to improve health [29,30]. The pre-clinical through phases II testing of this repeated implementation intention intervention have been completed as part of this previous work. In particular, significant work has been completed to develop the intervention and test its feasibility and acceptability in appropriate samples of schoolchildren (see [12]). In the control condition participants will read persuasive messages encouraging them to complete all their home work and they will form an implementation intention in relation to completing all their homework.

The intervention will take place in classroom time and will be led by a teacher using materials provided by the researchers. The materials will include written simple anti-smoking messages (as in our previous research, these will be varied between sessions to reduce boredom) and paper versions of the implementation intentions sheets to be completed. The time required will be no longer than 60 minutes to include an introduction by the teacher, brief classroom discussion, reading of the anti-smoking messages and completion of the paper and pencil implementation intention task. We will negotiate with schools about the least disruptive and most appropriate teaching session in which to implement these sessions (e.g., morning registration). Teachers will be provided with a one-to-two hour training session to introduce them to implementation intentions and anti-smoking messages, and to provide them with advice and written information on how to run the intervention sessions. This training will be conducted by the research team and teachers will be paid for their time. We will negotiate with schools on whether the same or different teachers will run the sessions throughout the trial and it may be necessary to train some additional teachers each year.

### Intervention fidelity

We will assess adherence to the intervention in two ways. First, approximately 10% of intervention sessions will be assessed for adherence to protocol (a mixture of self-report by teachers and observation by researchers). Second, all written implementation intentions sheets will be collected by teachers and passed to research staff and they will be analysed for completeness. In our previous research [8] 73% of adolescents completed implementation intentions sheets on at least 6 occasions (out of a total of 8 occasions). In addition, the number of occasions implementation intentions sheets were completed was unrelated to reduced smoking initiation. In the present trial the impact of number of complete and partially complete implementation intentions sheets on rates of objective and self-reported smoking will be examined statistically. Both sets of data will inform the basis of an intervention fidelity analysis.

### Analyses

#### Analyses of primary and secondary outcome measures

The study uses a two group experimental design with schools of adolescents (all aged 11–12 years at outset) randomly allocated to one of two conditions: a repeated implementation intention condition targeting smoking or a control condition. The primary outcome will be objectively assessed smoking and the secondary outcome measure will be self-reported smoking. A two-level logistic regression will be used to model smoking outcomes at 48 months. Given that drop outs are expected, multiple imputation, based on regression methods, will be undertaken to ‘complete’ the data and ensure efficient analysis. This will be done for all participants for whom baseline data is available (to provide meaningful imputations) and will take into consideration the two-level hierarchical nature of the data. With this approach the results will be unbiased given the general assumption of missing at random (MAR) rather than the more restrictive assumption of missing completely at random (MCAR). Results from the multiple imputations will be combined using Rubin’s rules.

Subgroup analyses will be used to examine any differences between high and low deprived schools, between regions (West Yorkshire versus Staffordshire), and between boys and girls. We will use mediation analyses to explore the extent to which any intervention effects are mediated by changes in the measured cognitions as tested in our previous work (e.g., attitudes to smoking, intentions to resist smoking, self-efficacy to not smoke, etc.; [31-34]).

#### Economic analyses

We will also conduct an economic evaluation of the intervention. The study will take the perspective of the service providers, health and social care sectors. Costs will include the resources used in service provision and will be collected using an adapted version of a form developed by the Health Economics Group at the University of Leeds for an on-going trial with a population of adolescents. Costs will also include intervention time, resources used on training and materials. First, the economic sub-study aims to identify the within study incremental cost effectiveness ratio (ICER); the costs and benefits of the intervention compared to the control group. The analysis will use smoking rates in the two arms of the trial, and ‘cost per non-initiation of smoking’ will be estimated. Second, it will adopt a lifetime horizon and cost per life year gained will be estimated from a decision analytic cost effectiveness model. As far as possible parameters in the model will be specified using data collected within the trial. Data on resource use costs and quality of life will be collected. Other parameters, such as the long term ‘natural history’ will be parameterised using the published literature, and where necessary formally elicited expert opinion. The outcome measure for these analyses will be life years gained [35]. The calculation of QALY’s will also be explored, using a measure of quality of life. In the analysis, the non-parametric bootstrap method will be used to produce a within trial probabilistic sensitivity analysis of the ICER. A scatterplot of the cost effectiveness plane, the 95% cost effectiveness elipse and cost effectiveness acceptability curve will be presented. Discounting will be undertaken using the rate recommended at the time.

## Discussion

Reducing smoking initiation is potentially the most effective way to reduce smoking-related harm. The present paper reports the protocol for an intervention designed to reduce smoking initiation in adolescents. The intervention involves the repeated formation of implementation intentions (i.e. if-then plans) about how to refuse offers of cigarettes Promising initial data have been collected on the efficacy of this intervention [8]. The current cluster randomised controlled trial will collect further data on the effectiveness of the intervention. This is a simple intervention that could be deployed across most schools (i.e., has wide reach) in order to help tackle smoking initiation in adolescents. In addition, this intervention is relatively low cost, requiring around 30-60 minutes per session to implement, and can be implemented by teachers. Should the intervention prove effective in changing smoking outcomes and prove ‘value for money’ it might usefully be rolled out to significant proportions of the adolescent population in the UK.

## Competing interests

The authors declare that they have no competing interests.

## Authors’ contributions

MC conceived the study. MC, SG, RL, CA, RW, KS, BG and CT were applicants for the funding and involved in designing the study. MC led the drafting of the protocol with input from all authors. All authors read and approved the final protocol.

## Pre-publication history

The pre-publication history for this paper can be accessed here:

http://www.biomedcentral.com/1471-2458/13/54/prepub
